# Agoraphobia and panic attacks complicated by primary aldosteronism improved by treatment with eplerenone: a case report

**DOI:** 10.1186/s12888-023-05275-w

**Published:** 2023-10-27

**Authors:** Reoto Kijima, Hirofumi Tesen, Ryohei Igata, Naomichi Okamoto, Reiji Yoshimura

**Affiliations:** Department of Psychiatry, University of Occupational and Environmental Health, 1-1 Iseigaoka, Yahatanishi-ku, 8078555 Kitakyushu, Fukuoka Japan

**Keywords:** Agoraphobia, Panic Attacks, Primary aldosteronism, Mineralocorticoid

## Abstract

**Background:**

Primary aldosteronism (PA) is an adrenal gland disease, that induces increased secretion of the mineralocorticoid, aldosterone, resulting in symptoms such as hypertension. This study reports a patient with agoraphobia and panic attacks, associated with PA. This patient’s psychiatric symptoms improved after treatment with eplerenone, a mineralocorticoid receptor antagonist.

**Case presentation:**

The patient was a 40-year-old female with agoraphobia, which refers to the irrational fear of situations that may cause anxiety, and panic attacks characterized by profuse sweating, palpitations, and generalized weakness. She was diagnosed with hypertension from PA. Subsequently, she received treatment with eplerenone, which improved her agoraphobia and panic attacks.

**Conclusions:**

There have been no previous reports on PA associated with agoraphobia and panic attacks that improved with pharmacotherapy. Patients with agoraphobia and panic attacks should be evaluated for PA. In patients with PA, pharmacotherapy with eplerenone should be considered.

## Background

Primary aldosteronism (PA) is an adrenal gland disorder, that causes overproduction of the mineralocorticoid, aldosterone, resulting in hypertension. PA can be attributed to approximately 1% of all hypertensive patients, but in recent years it has been estimated to be 5–10% [1]. Unilateral PA secondary to an adrenal adenoma is treated via unilateral adrenalectomy, while bilateral PA secondary to adrenal hyperplasia is treated with mineralocorticoid receptor (MR) antagonists. Patients unwilling to undergo surgery also receive MR antagonists. The complications of PA include depression, anxiety disorders, and other psychiatric disorders [2–5]. There have been some reports of PA, complicated with agoraphobia or panic attacks [5, 6]. However, this was the first study to document the resolution of agoraphobia and panic attacks in a patient with PA, who was treated pharmacologically. This study reports the case of a patient with agoraphobia and panic attacks induced by PA. The patient’s psychiatric symptoms improved after treatment with eplerenone, an MR antagonist.

## Case presentation

The patient was a 40-year-old female with a chief complaint of physical immobility. She didn’t have genetic factors, associated with neuropsychiatric diseases. After graduating from high school, she studied abroad at a University College. After graduating from the University, she returned to her hometown and worked in sales and business. She developed uterine cancer and complete remission was achieved postoperatively. She did not have a history of mental illness. Four months prior to her visit to our university hospital, she was at the company’s training center, where she experienced sweating, palpitations, breathlessness, lightheadedness, and weakness. Her symptoms made it difficult to move her extremities. These symptoms were repeatedly observed in similar situations. Her symptoms improved when she moved away from the training center. She began experiencing similar symptoms during online meetings. She consulted with her local physician, who recorded a systolic blood pressure of at least 180 mmHg, plasma aldosterone concentration (PAC) of 69.6 pg/mL (CLEIA method), active renin quantitative concentration (ARC) of 1.4 pg/mL (CLEAR method), and positive PAC/ARC 50 and PA screening tests. She was admitted to the Internal Medicine Department of the University Hospital. She was seen by a hospital physician, and similar symptoms were elicited. Since her attacks were precipitated by specific situations and improved upon avoiding these triggers, a psychiatric disorder was suspected. Thus, the patient was referred to the Department of Psychiatry. The patient’s facial expressions and tone of voice were calm and composed. She experienced attacks in certain situations such as when she entered the company’s training center or during online meetings. During the attacks, she was aware of her lightheadedness, weakness of the upper and lower extremities, inability to stand up for an extended period, palpitations, sweating, and some difficulty in breathing. These attacks frequently occurred when she went to the company’s training centers and online meetings. In the hospital, the same symptoms were observed, when she was in the examination room with a physician. The symptoms lasted for several minutes and improved when the patient moved away from the trigger. She became anxious about these situations and eventually developed an avoidant behavior. The symptoms occurred when the patient went to training center, online meetings, consultations with the doctor, and the examination room. The total score of the Japanese version of the self-rating Panic and Square Fear Scale (PAS) [7] was 26. The symptoms were possibly caused by PA. However, they were triggered by specific situations and improved upon avoiding these triggers. Moreover, the patient’s weakness was not caused by a documented decrease in her potassium level. Thus, the symptoms were not solely caused by PA. Rather, her symptoms constituted a panic attack. The patient experienced fear and anxiety, when she was placed in the aforementioned situations, and she tended to avoid them. Her symptoms were triggered by situations involving social interactions despite having minimal fear of attracting attention from others. Based on this, a social anxiety disorder was less likely, and the patient was diagnosed with agoraphobia. A positive captopril stress test and furosemide standing test were performed to identify the cause of hypertension, and the patient was diagnosed with PA. Abdominal computed tomography showed no adrenal gland tumors, and elective adrenal vein sampling was considered to determine if surgery was indicated. However, the patient did not wish to undergo surgery. Hence, she received pharmacological treatment. She was given eplerenone (25 mg), an MR antagonist. Five days later, the dosage was increased to 50 mg. After initiating eplerenone therapy, the patient’s panic attacks persisted, albeit less severely. The panic attacks were associated with a feeling of chest heaviness and the duration of the attacks was reduced. Based on this, the treatment improved the patient’s agoraphobia and panic attacks. She was discharged Day22 after admission. Following her discharge, her psychiatric symptoms gradually improved. She still had panic attacks upon entering the training center and during medical examinations. Subsequently, she was prescribed alprazolam (0.4 mg), but it was discontinued after a single dose due to somnolence. Her blood pressure remained at 140/90 mmHg. The dosage of her eplerenone was increased to 75 mg. Four days after the increase dosage, her psychiatric symptoms disappeared, and she has not experienced a panic attack or agoraphobia since then.

## Discussion and conclusions

This was the first case of PA-induced agoraphobia and panic attacks, that were completely resolved with eplerenone treatment. Several reports have shown the relationship between PA, psychiatric disorders, and psychiatric symptoms. Among ten patients with PA, six had generalized anxiety disorder. One patient had panic disorder, while another had major depressive disorder [4]. The frequency of psychiatric disorders, stress, mental status, and well-being were assessed and compared among three groups: 23 patients with PA, 23 patients with essential hypertension, and 23 normotensive healthy controls. The prevalence of anxiety disorders was significantly higher in the PA group, compared to the other two groups [5]. One patient in this study had agoraphobia, complicated by PA, and reports of PA complicated by agoraphobia were limited to this case. Although the mechanism behind the development of psychiatric complications in PA patients has not been elucidated, animal experiments have reported that MR, a receptor for aldosterone, was also expressed in the hippocampus and amygdala, which control anxiety and fear [8, 9]. Chronic aldosterone administration reportedly increased anxiety-like behavior in rats [10]. In the present case, the onset of the patient’s agoraphobia and panic attacks was consistent with the development of hypertension from PA. Based on this, PA possibly induced her agoraphobia and panic attacks. The gradual decrease and eventual disappearance of psychiatric symptoms with eplerenone, an MR antagonist, also supported the association between PA and her agoraphobia and panic attacks.

Eplerenone is an MR antagonist, that selectively inhibits MR. It is used to treat hypertension. Unilateral PA secondary to an adrenal adenoma is treated via unilateral adrenalectomy, while bilateral PA due to adrenal hyperplasia is treated with MR antagonists, such as eplerenone. Pharmacotherapy is used when surgery is not required. Based on rat experiments, the chronic administration of eplerenone induced anxiolytic effects [11]. In a previous report, a patient with PA, complicated by panic attacks, received treatment with spironolactone, another MR antagonist. The patient’s symptoms gradually improved with pharmacological treatment and significantly enhanced after undergoing an adrenal adenomectomy [6]. In our study, the patient was not desirous of surgery, so oral eplerenone was prescribed. The patient’s agoraphobia and panic attacks improved with oral MR antagonist treatment, and the symptoms eventually disappeared. There have been no reports, documenting the complete resolution of PA-associated agoraphobia and panic attacks with oral MR antagonist treatment alone.

This study reports a case of PA, associated with agoraphobia and panic attacks, that improved with eplerenone. Based on the results of this study, eplerenone was an effective treatment option for PA-associated agoraphobia and panic attacks among patients not desirous of surgery.

## Data Availability

The data supporting the findings of this report are available from the corresponding author upon reasonable request.
